# Supplementary material for: Multi‐dataset identification of innovative feature genes and molecular mechanisms in keratoconus
Source: J Cell Mol Med. 2024 Sep 19;28(18):e70079. doi: 10.1111/jcmm.70079 (PMC11412914; doi:10.1111/jcmm.70079)
Supplement: Supplementary file 2 — Material S2. [file JCMM-28-e70079-s002.zip › 7.GSEA analysis/3.DBNDD1/KEGG_COLORECTAL_CANCER.html]

Details for gene set KEGG\_COLORECTAL\_CANCER[GSEA]

|  || Dataset | DBNDD1.group.cls#h\_versus\_l |
| Phenotype | group.cls#h\_versus\_l |
| Upregulated in class | h |
| GeneSet | KEGG\_COLORECTAL\_CANCER |
| Enrichment Score (ES) | 0.21085954 |
| Normalized Enrichment Score (NES) | 0.84821284 |
| Nominal p-value | 0.6666667 |
| FDR q-value | 1.0 |
| FWER p-Value | 1.0 |
Table: GSEA Results Summary

  

Fig 1: Enrichment plot: KEGG\_COLORECTAL\_CANCER      
 Profile of the Running ES Score & Positions of GeneSet Members on the Rank Ordered List

  

| SYMBOL | TITLE | RANK IN GENE LIST | RANK METRIC SCORE | RUNNING ES | CORE ENRICHMENT || 1 | PIK3R5 | na | 35 | 0.708 | 0.1354 | Yes |
| 2 | DCC | na | 1372 | 0.347 | 0.1637 | Yes |
| 3 | RAC3 | na | 1824 | 0.312 | 0.2109 | Yes |
| 4 | TGFBR2 | na | 4515 | 0.197 | 0.1712 | No |
| 5 | PIK3R1 | na | 6912 | 0.126 | 0.1264 | No |
| 6 | SMAD2 | na | 7383 | 0.116 | 0.1352 | No |
| 7 | AKT2 | na | 9388 | 0.080 | 0.0928 | No |
| 8 | CCND1 | na | 9676 | 0.076 | 0.0991 | No |
| 9 | MAPK3 | na | 9865 | 0.073 | 0.1078 | No |
| 10 | MAPK10 | na | 10497 | 0.065 | 0.1021 | No |
| 11 | APPL1 | na | 10995 | 0.058 | 0.0990 | No |
| 12 | MAPK9 | na | 11537 | 0.052 | 0.0933 | No |
| 13 | PIK3R2 | na | 11699 | 0.050 | 0.0983 | No |
| 14 | SMAD4 | na | 11961 | 0.047 | 0.1000 | No |
| 15 | CASP3 | na | 13339 | 0.035 | 0.0671 | No |
| 16 | FOS | na | 13400 | 0.035 | 0.0721 | No |
| 17 | BAX | na | 13486 | 0.034 | 0.0762 | No |
| 18 | TGFB3 | na | 13624 | 0.033 | 0.0787 | No |
| 19 | TP53 | na | 13962 | 0.030 | 0.0748 | No |
| 20 | KRAS | na | 14127 | 0.029 | 0.0756 | No |
| 21 | MSH3 | na | 14141 | 0.029 | 0.0808 | No |
| 22 | PIK3R3 | na | 14144 | 0.029 | 0.0863 | No |
| 23 | RALGDS | na | 14647 | 0.025 | 0.0767 | No |
| 24 | RAC1 | na | 14759 | 0.025 | 0.0783 | No |
| 25 | BRAF | na | 15471 | 0.019 | 0.0615 | No |
| 26 | CTNNB1 | na | 16378 | 0.013 | 0.0380 | No |
| 27 | PIK3CA | na | 17073 | 0.009 | 0.0196 | No |
| 28 | PIK3CB | na | 18338 | 0.000 | -0.0167 | No |
| 29 | RAF1 | na | 19005 | -0.005 | -0.0351 | No |
| 30 | AKT1 | na | 19066 | -0.005 | -0.0358 | No |
| 31 | RHOA | na | 19096 | -0.005 | -0.0357 | No |
| 32 | TGFB2 | na | 19303 | -0.007 | -0.0403 | No |
| 33 | AXIN1 | na | 19524 | -0.009 | -0.0449 | No |
| 34 | MSH2 | na | 19838 | -0.012 | -0.0517 | No |
| 35 | CASP9 | na | 19881 | -0.012 | -0.0507 | No |
| 36 | SMAD3 | na | 19999 | -0.013 | -0.0515 | No |
| 37 | TCF7L2 | na | 20073 | -0.014 | -0.0510 | No |
| 38 | CYCS | na | 20083 | -0.014 | -0.0486 | No |
| 39 | BAD | na | 20453 | -0.017 | -0.0560 | No |
| 40 | RAC2 | na | 20865 | -0.021 | -0.0638 | No |
| 41 | BIRC5 | na | 21430 | -0.026 | -0.0751 | No |
| 42 | APC2 | na | 21518 | -0.027 | -0.0724 | No |
| 43 | GSK3B | na | 22452 | -0.037 | -0.0922 | No |
| 44 | MAPK8 | na | 22620 | -0.039 | -0.0896 | No |
| 45 | MYC | na | 22660 | -0.039 | -0.0832 | No |
| 46 | MAPK1 | na | 22705 | -0.040 | -0.0768 | No |
| 47 | MSH6 | na | 23138 | -0.045 | -0.0806 | No |
| 48 | MLH1 | na | 23263 | -0.046 | -0.0752 | No |
| 49 | TCF7 | na | 23571 | -0.051 | -0.0743 | No |
| 50 | JUN | na | 24077 | -0.057 | -0.0778 | No |
| 51 | APC | na | 24380 | -0.062 | -0.0747 | No |
| 52 | ARAF | na | 24817 | -0.068 | -0.0741 | No |
| 53 | PIK3CD | na | 24971 | -0.070 | -0.0650 | No |
| 54 | TGFBR1 | na | 26390 | -0.095 | -0.0875 | No |
| 55 | TCF7L1 | na | 26813 | -0.104 | -0.0797 | No |
| 56 | PIK3CG | na | 27455 | -0.118 | -0.0755 | No |
| 57 | AXIN2 | na | 28854 | -0.153 | -0.0863 | No |
| 58 | MAP2K1 | na | 29246 | -0.164 | -0.0660 | No |
| 59 | TGFB1 | na | 30914 | -0.218 | -0.0721 | No |
| 60 | BCL2 | na | 31549 | -0.242 | -0.0437 | No |
| 61 | LEF1 | na | 32857 | -0.317 | -0.0202 | No |
| 62 | AKT3 | na | 33656 | -0.385 | 0.0309 | No |
Table: GSEA details [plain text format]

  

Fig 2: KEGG\_COLORECTAL\_CANCER      
 Blue-Pink O' Gram in the Space of the Analyzed GeneSet

  

Fig 3: KEGG\_COLORECTAL\_CANCER: Random ES distribution      
 Gene set null distribution of ES for **KEGG\_COLORECTAL\_CANCER**
